# Lifetime Employment Trajectories and Cancer: A Population-Based Cohort Study

**DOI:** 10.21203/rs.3.rs-4207039/v1

**Published:** 2024-04-15

**Authors:** Stéphane Cullati, Stefan Sieber, Rainer Gabriel, Matthias Studer, Arnaud Chiolero, Bernadette Wilhelmina Antonia van der Linden

**Affiliations:** University of Fribourg; Barcelona Institute for Global Health; Zurich University of Applied Sciences; University of Geneva; University of Fribourg; University of Fribourg

**Keywords:** Cancer, Employment, Epidemiology, Life Course, Sequence Analysis, Social determinants of health

## Abstract

Working life is associated with lifestyle, screening uptake, and occupational health risks that may explain differences in cancer onset. To better understand the association between working life and cancer risk, we need to account for the entire employment history. We investigated whether lifetime employment trajectories are associated with cancer risk. We used data from 6,809 women and 5,716 men, average age 70 years, from the Survey of Health, Ageing, and Retirement in Europe. Employment history from age 16 to 65 was collected retrospectively using a life calendar and trajectories were constructed using sequence analysis. Associations between employment trajectories and self-reported cancer were assessed using logistic regression. We identified eight employment trajectories for women and two for men. Among women, the risk of cancer was higher in the trajectories “Mainly full-time to home/family”, “Full-time or home/family to part-time”, “Mainly full-time”, and “Other” compared with the “Mainly home/family” trajectory. Among men, the risk of cancer was lower in the “Mainly self-employment” trajectory compared with “Mainly full-time”. We could show how employment trajectories were associated with cancer risk, underlining the potential of sequence analysis for life course epidemiology. More research is needed to understand these associations and determine if causal relationships exist.

## Introduction

Cancer is associated with early life course influences, such as socioeconomic circumstances^1–5^ and quality of parenting^6^ in childhood, or main occupation in early adulthood^7^ and middle age^8,9^. There are many association studies correlating cancer with socioeconomic circumstances at a given moment in the life course, such as childhood^10^, or with two or three life course moments^3,7,11,12^, but little is known about how entire life course trajectories (with information on each year of life) can predict the onset of cancer.

Life course epidemiology, and the study of social determinants of health, have shown that where people work and live throughout the life course^13^ shapes their health and how they adapt to stress and age^14–17^. Cancer determinants research is interested in understanding how these conditions throughout the life course may influence the early development of cancer^18,19^. One set of conditions in particular, our working lives, is central to our life course as we spend a large proportion of our time at work^20^. Exposure to work throughout life therefore has both positive and negative effects on health in later in life^21–24^, and may be associated with the development of cancer^25–30^. Previous studies have shown that past labour market status (hereafter, *employment status*) is associated with health in old age^31–51^ and cancer^8,52^. However, these studies have focused on one-off indicators of current or past employment status, which do not take into account the complex nature of individual work histories.

Another approach to assessing the influence of the life course on health in old age is to use the information on the whole history of the participanťs life course, for example from adolescence to the middle age (or the beginning of old age), collected through life history interviews^53^. Using this approach, studies have described employment trajectories over the life course^54–57^, with women’s trajectories being more diverse than those of men^54^. Other studies have shown that employment trajectories are associated with quality of life^58,59^, self-rated health^31,60,61^, mental health^58,60,62–68^, and functional health^66,69^, but no study has investigated a possible association with cancer over the life course.

We hypothesise, therefore, that cancer onset in old age is associated with employment trajectories over the life course. Using data from a large population-based cohort study, we first described the men’s and women’s employment history from 16 to 65 years of age by classifying them into types of trajectories using sequence analysis; second, we analysed the associations between men’s and women’s employment trajectories and cancer onset over the life course. For exploratory purposes, we also examined the associations with breast cancer.

## Results

### Participant characteristics

**Figure 1:**
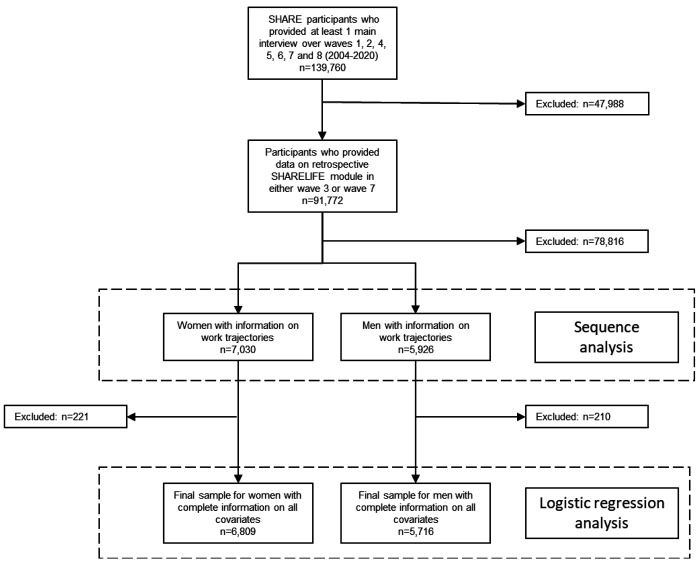
Flow chart of respondents’ inclusion

The flow chart (Fig. 1) describes the exclusion of participants to obtain the analytical sample for this study. First, we excluded participants who did not participate in the SHARELIFE module (n = 47,988 excluded). Second, we excluded participants who did not provide any information on their work or employment history between the ages of 16 to 65 (n = 78,816 excluded). After excluding participants with missing information on covariates, the final analytic sample consisted of 6,809 women and 5,716 men living in 14 European countries (Austria, Belgium, Czech Republic, Denmark, France, Germany, Greece, Ireland, Italy, Netherlands, Poland, Spain, Sweden, Switzerland). Women and men participated in an average of 5 waves (median 5, minimum 2, maximum 8).

The mean age at baseline was 70.7 years (SD 7.0) for women without diagnosed cancer and 70.1 years (6.7) for women with diagnosed cancer. Mean age was similar in men. The mean BMI was 26.5 in women and 26.8 in men, and the mean BMI was similar in participants with and without cancer in both genders. The majority of women and men were non-smokers and were physically active (i.e., engaged in any activity more than once a week). The proportion of women with fewer than two chronic conditions was slightly higher in the group without cancer (52.8%) than in the group with cancer (46.2%); this difference was slightly smaller in men: 62.4% in the group without cancer and 57.4% in the group with cancer.

A total of 781 women and 827 men were diagnosed with cancer at any time during their lifetime (Table 1). The distribution of age at cancer diagnosis is reported in Figures S1 and S2 (Supplementary material). 31.0% of women and 17.9% of men were diagnosed before the age of 65.

### Employment trajectories of women: sequence analysis

**Figure 2:**
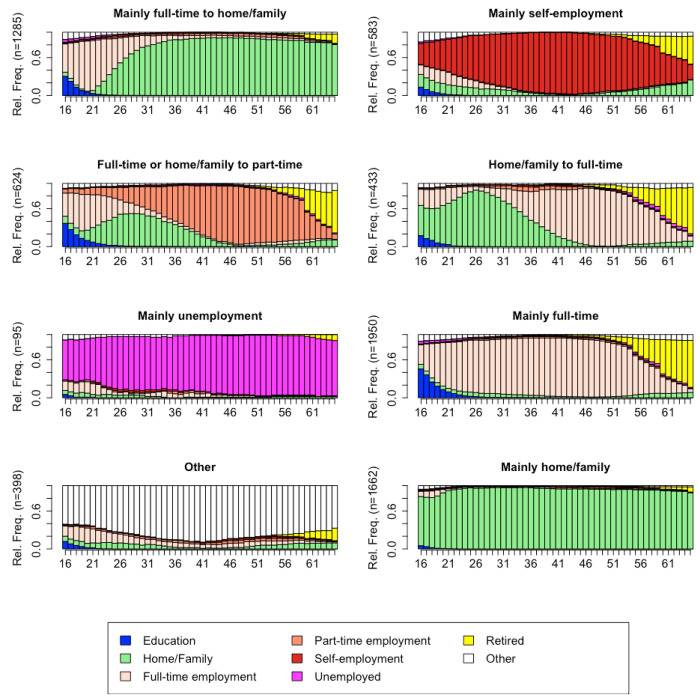
Chronogram of the eight employment trajectories for women aged 16-65. Data from the Survey of Health, Ageing, and Retirement in Europe (SHARE). Average silhouette width of 0.34.

For women, a solution with 8 employment trajectories (clusters) was chosen (Fig. 2). Here, the ASW of 0.34 indicated that the resulting clustering solution was of reasonable quality. The largest employment trajectory regrouped women who worked mainly full-time throughout their working lives (n = 1,950, 27.7%, this group was named “Mainly full-time”). This group of women also spent some time in education during adolescence. The second largest employment trajectory grouped women who were mainly involved in home and family, with very little time either in education or in the labour market (n = 1,662, 23.6%, “Mainly home/family”). Another employment trajectory (n = 1,285, 18.3%) included women who moved from full-time work to home / family (“Mainly full-time to home/family”). This group of women also spent some time in education during adolescence. These first three groups represented more than 70% of the sample, while the remaining five groups each represented less than 10% of the sample. Among the last five groups, we found one employment trajectory (n = 583, 8.3%) that included women who were mainly self-employed during their working life (“Mainly self-employment”). Another employment trajectory (n = 624, 8.9%) included women who started their working lives in either full-time or part-time employment, with a significant proportion moving to home/family in their twenties, and then moving to mainly part-time employment (“Full-time or home/family to part time”). A small number of women (n = 433, 6.2%) had an employment trajectory in which they spent the first half of their working lives mainly at home and then worked full-time in the second half of their working lives (“Home/Family to full-time”). An even smaller group of women (n = 95, 1.4%) were mainly unemployed during their working lives (“Mainly unemployed”). 398 women (5.7%) had an employment trajectory mostly in the “other” category, indicating more unusual trajectories characterised by illness or disability, voluntary work, and travelling (“other”).

### Employment trajectories of men: sequence analysis

**Figure 3:**
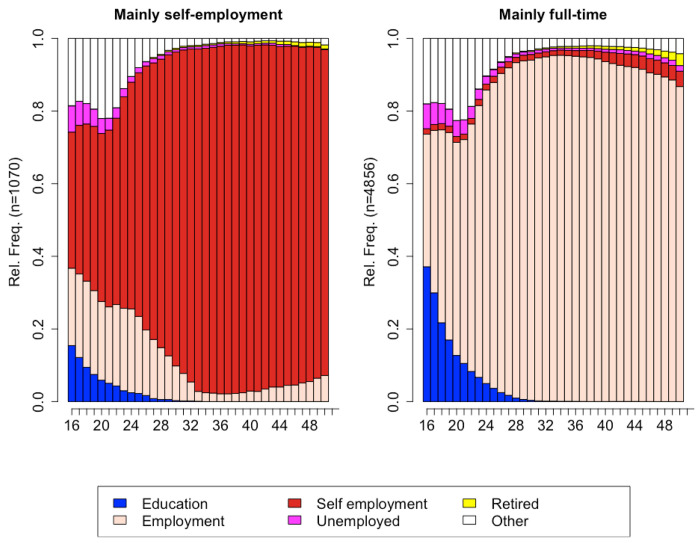
Chronogram of the two employment trajectories for men aged 16-65. Data from the Survey of Health, Ageing, and Retirement in Europe (SHARE). Average silhouette width of 0.70.

For men, a solution with two clusters was retained based on an ASW of 0.70, indicating a strong clustering structure (Fig. 3). The first employment trajectory included men whose working life was mainly characterised by self-employment (n = 1,070). The second employment trajectory included men (n = 4,856) who spent most of their working lives in full-time employment.

### Associations of women’s employment trajectories with cancer

Table 2 shows the results of the logistic regression analyses. After adjustment with covariates and attrition, compared with women in the “Mainly home/family” employment trajectory (reference category), women in the “Mainly full-time to home/family” trajectory, the “Full-time or home/family to part-time” trajectory, the “Mainly full-time” trajectory, and the “Other” trajectory had a higher risk of overall cancer (odds ratios (ORs) of 1.51, 2.28, 1.73, and 1.47, respectively).

Results for breast cancer are reported in the supplementary appendix (Table S1). The association between types of employment trajectories and breast cancer risk was similar to that for overall cancer, but with slightly higher ORs (1.68, 2.38, 1.92, 1.79, respectively).

### Associations of men’s employment trajectories with cancer

After adjustment with covariates and attrition, compared with men in the “Mainly full-time” employment trajectory (reference category), those in the “Mainly self-employment” trajectory had a lower risk of overall cancer (OR 0.74 95%CI 0.60–0.90).

## Discussion

In this study, we first described the employment trajectories from age 16 to 65 of a cohort of Europeans born between 1914 and 1945, and second, we assessed whether these employment trajectories could predict the onset of cancer at any time in the life course. First, using sequence analysis, we grouped the employment histories from the ages of 16 to 65 into eight and two patterned trajectories for women and men, respectively. For women, we named the eight trajectories “Mainly full-time”, “Mainly home/family”, “Mainly full-time to home/family”, “Mainly self-employment”, “Full-time or home/family to part time”, “Home/family to full-time”, “Mainly unemployment” and “Other”. In terms of the proportions of the sample, none of the eight trajectories was in the majority. In this “prediction exercise” ^70^, and thanks to sequence analysis, we found that, compared to women who spent their lives mainly caring for the home/family, four employment trajectories were associated with a higher risk of cancer over the life course: women who worked predominantly full-time, women who worked full-time then transitioned to taking care of home and the family, women who either worked full-time or took care of the home/the family and then transitioned to working part time, and women with unusual employment trajectories (“Other”), i.e. trajectories characterised by illness or disability, voluntary work, and travelling. When we looked at breast cancer, we also found associations with the same four employment trajectories. For men, we named the two solutions “Mainly full-time” and “Mainly self-employment”, with the first one representing the largest, majority group. In sum, this study has shown that the onset of cancer over the life course can be predicted by classifying women and men into typical groups of lifetime employment trajectory, and that these associations were independent of factors known to be associated with cancer (smoking, physical activity, number of chronic conditions, and body mass index).

The finding of eight employment trajectories for women suggested that women’s employment trajectories were more heterogeneous, or less “socially standardised” by the culture and local contexts, than men’s, who had only two trajectories, with one predominant type being full-time work throughout their working lives. The heterogeneity of women’s employment trajectories and the homogeneity of men’s employment trajectories are consistent with previous studies in Germany^56^, England^71^ and Switzerland^54^, and, at least for women, with previous studies using the same dataset^59,72^. However, using the same dataset, another study found two patterned employment trajectories for women, but using multi-channel sequence analysis^73^. The finding of heterogeneity in women’s employment trajectories was to be expected for these cohorts as women’s life course trajectories, compared with men’s, are socially normed towards multiple social roles, such as parenthood, family responsibilities, including contribution to breadwinning, and – to a lesser extent – professional career^74–77^.

Individuals’ employment trajectories are influenced by the policies of the country in which they live^78,79^. Indeed, policies can affect employment opportunities, job security, wages and career paths. For example, labour market policies implemented by governments (such as employment protection legislation, labour market flexibility measures, reintegration measures), social security and welfare policies (such as unemployment benefits, income support programmes), family policies (childbearing) and health policies (health insurance, disability insurance) can have direct or indirect effects on employment trajectories. Such effects were not investigated in this study but it could be assumed that country policies might moderate some of the associations observed in this study. Such research may prove challenging as it would require historical research on labour market, social security, family and health policies in each of the countries included for the period covering the participants’ employment trajectories.

The diversity of employment trajectories was associated with higher cancer risk over the life course. In our study, we identified several groups of lifetime employment trajectories in which work in general (either full-time or part-time) was associated with cancer in women. We found that a lifetime trajectory of working full-time as an employee was associated with a higher risk of cancer compared with a lifetime trajectory of caring for home and family. In addition, working full-time as an employee in the first half of the working life and then transitioning into the family sphere was also associated with a higher risk of cancer. There are three possible interpretations of these findings. First, given that the association with cancer reflects breast cancer risk, it is possible that women who entered the labour market at some point in their life course were more likely to be informed and screened for breast cancer, and therefore had more cancer diagnoses. A European cross-sectional study suggested that being inactive was associated with a lower likelihood of being up to date with cancer screening^80^. Second, if full-time employment does increase women’s cancer risk, it is not *per se*, but because employment may be an indirect marker of the poor quality of women’s jobs and of occupational stress. Indeed, in the second half of the twentieth century women were known to have a higher proportion of low-skilled jobs^81^. Women from these cohorts increased their participation in the labour market but were more likely to be employed in manual occupations and in low-value jobs^82^. It is also possible that employed women are more exposed to occupational stress than women who have been self-employed throughout their working lives. Work can expose individuals to social and psychological stressors^83^, and stress has a biopsychosocial pathway to cancer development^30,84–86^. Gender insensitive working environments may affect mental health^87^, which may also predict cancer incidence through depression and psychological distress^85^. Exposure to more or less long-term poor working conditions^88^ may dysregulate physiological systems (e.g., immunity^89,90^), or the general physiological wear and tear^19^ and DNA methylation^91,92^, and predict the onset of cancer^93,94^. It is likely that the employment status – the indicator on which our sequence analysis was based - is an imperfect marker of the resources (income, job-related skills, social network at work) and status (social status of occupations) that women derive from their occupations and from the types of responsibilities they have at work, and ultimately of the levels of stress to which they are exposed^95,96^. Thus, without lifetime data on occupation and on psychosocial stress at work (e.g., effort-reward balance), the association between employment exposure and increased cancer risk should be treated with great caution.

Among men, we observed an association between a lifetime trajectory of self-employment and cancer risk. Compared with men who worked full-time as employees throughout their working lives, those who were self-employed had a lower risk of cancer. There is little research on the relationship between self-employment and better health for men. Self-employed people may generally have better health compared to employees for the following reasons: firstly, self-employed men may have more control over their work environment and workload, and therefore be better able to manage the stress levels; secondly, self-employed men may have more flexibility in their working hours, which may allow them to engage in healthy behaviours such as exercise and healthy eating, or cancer screening. On the other hand, we cannot ignore that the self-employed are exposed to the risks and responsibilities that come with running a business, which can lead to stress^97,98^, thus limiting the proposed explanation.

This study has limitations. First, we cannot exclude that reporting bias on the outcome may be significant, for two reasons: (a) cancer diagnosis was self-reported rather than from cancer registries. Self-reported cancer has an overall false-negative rate of 39.2%, with wide variation by cancer site^99^; (b) respondents in this cohort were older, which may be associated with more frequent false-positive reporting^100^. Second, survivor bias is inherent in the design of ageing studies such as SHARE, whose baseline included participants aged 50 years and over. However, the survivor bias may be limited by the low probability of dying from cancer before the age of 50^101^. Third, as shown in the flowchart of responden’s inclusion, the analytical sample (6,809 + 5,716 = 12,525) was a reduction compared to the original dataset (n = 139,760), signifying that selection bias may affect the results. Fourth, due to the lack of data in SHARE, our study does not take into account other life course factors such as intrauterine life, perinatal characteristics, child growth, and hormonal factors, which are associated with the incidence of several cancers^5,102^. Fifth, like all longitudinal studies of ageing, SHARE is characterised by attrition at follow-up^103^, and we have partially controlled for this in the analysis. Sixth, by controlling for cancer risk factors such as smoking^104^, sedentary behaviour^105^ and BMI^106,107^ measured at baseline, we confirmed that employment trajectories were independent of these risk factors, however we do not have information about the individual life course history of these factors, which may interact with employment over the working life. Seventh, the eight trajectories are not a definitive solution for categorising women’s trajectories because the clustering methods impose numerous choices, such as the clustering algorithm and the distance metric, which could influence the results. The eight trajectories are the result of a data mining construction that seeks the best solution for grouping the great heterogeneity of respondents’ trajectories (each individual sequence), taking into account the importance of the change (its timing and its order in the sequence) as well as the duration of a state (no change) in the employment trajectory. Eighth, the decisions made by SHARE about the coding of the employment status in 8 categories are critical in sequence analysis, as it will deeply influence the structure of the sequences. Ninth, the use of the life calendar allowed the reconstruction of the entire life course, retrospectively, on a yearly basis, between the ages of 16 and 65. Such a tool is useful for collecting comprehensive information on the life course of respondents. However, life calendars are subject to mnemonic and cognitive biases, but these are limited by the instrumen’s ability to enhance responden’s memory and correct for inconsistencies compared to a list of standardised questions^108^. Tenth, this study was based on participants who spent most of their lives between the ages of 16 and 65 in the 20th century, a context marked by gender inequalities in access to education and the labour market, which is assumed to influence the ageing of women in good health^109^. Due to changes in the social stratification and in social norms^110^, in particular progress in socioeconomic equality between women and men^81,111^, we expect that our findings may not be applicable to cohorts who will spend their life course in the 21st century. Eleventh, this study examined employment trajectories cross-nationally by merging respondents from 14 countries. Our approach should not mask large cross-national differences in employment trajectories^78,79^. Twelfth, the timing of exposure (employment trajectories from 16 to 65 years) overlapped with the onset of the outcome (cancer diagnoses from 0 to 99 years), with about three in ten female cancer diagnoses occurring before the age of 65 years.

Lifetime employment trajectories of European women are diverse and some of these trajectories were associated with a higher risk of self-reported cancer over the life course if women participated in the labour market compared with women who stayed at home. Lifetime employment trajectories of European men were less diverse, and being self-employed throughout working life was associated with a lower risk of cancer.

We believe that examining the full history of the participan’s life course using life calendar data and identifying patterned trajectories in these data using sequence analysis, has a promising potential for studies in life course epidemiology, although it has been rather rare to date in the field of epidemiology and health studies^59,71,112^. Although the lifetime employment trajectories approach looks promising, our association study remained a predictive exercise^70^. More research is needed to better understand these associations and to establish whether they are causal. In particular, factors such as health-related behaviours and occupational health risks, and their timing over the life course, should be considered in further studies.

## Methods

### Study design and population

The Survey of Health, Ageing, and Retirement in Europe (SHARE) is an ongoing longitudinal and cross-national survey designed to investigate population ageing processes and includes individuals aged 50 years and older^113^. SHARE was approved by the Ethics Council of the Max Planck Society and the relevant national research ethics committees in the participating countries, confirming that the survey was conducted in accordance with the Declaration of Helsinki as well as all relevant legal and ethical guidelines and regulations. All participants provided written informed consent.

Currently, SHARE includes eight waves of data that were collected every two years between 2004 and 2020 from 28 countries (12 countries in 2004). In the third and seventh wave, retrospective life history data were collected in the SHARELIFE module on past work or employment history from age 16 to 65 using a life calendar^114^.

### Employment trajectories

The analysis of employment trajectories utilized the Harmonized Share Life History Dataset, Version B, February 2020, provided by the Gateway to Global Aging Data. Only individuals who had information on employment trajectories until the age of 65 were retained. The data was then split based on gender (variable “ragender”) and the clustering of trajectories was performed on each gender-specific dataset.

The analyses of employment trajectories are based on the variable “workstate” which is available from the ages 15–80. This variable describes the labour market status of the participants and is constructed using available information on paid work (as an employee or self-employed), unpaid work (domestic work or family work), or whether a person was not working (education, episodes of illness, etc.). Thus, for each year, the employment status of the participant is characterised according to one of these eight situations: 1. Employed full-time, 2. Employed part-time, 3. Self-employed, 4. Unemployed, 5. Home/family, 6. Retired, 7. Full-time education, and 8. Other. The “Other” category includes: Illness or disability, voluntary work, military service, and travelling.

### Cancer

The question “Has a doctor ever told you that you had/Do you currently have any of the conditions on this card?” was used to operationalize cancer^1^. Participants who selected the ‘Cancer: ever diagnosed/currently having’ option were included in the analyses as having cancer. This included participants whose doctor had told the participants that they had cancer and who were currently being treated for or bothered by cancer. The follow-up question on specific cancer sites was used to run the analyses by site.

### Covariates

The analyses were adjusted for attrition (no dropout, dropout, deceased) and covariates. The latter includes birth cohort, body mass index (BMI), smoking status, chronic conditions and physical activity levels. Birth cohort was categorized as born not during a crisis or war period (i.e., born before 1914, between 1919 and 1928, or after 1945), born during a war period (i.e., born between 1914 and 1918 or between 1939 and 1945), or born during the Great Depression (i.e., born between 1929 and 1938). BMI was derived using self-reported weight and height at baseline. Smoking status at baseline was used to categorize participants into non-smokers or smokers. The number of chronic conditions was calculated based on the following conditions at baseline and then transformed into a binary variable indicating (0) less or (1) two or more chronic conditions: stroke, heart attack, hypertension, high blood cholesterol, diabetes, chronic lung disease, stomach or duodenal ulcer, peptic ulcer, Parkinson disease, Alzheimer’s disease, affective or emotional disorders, including anxiety, nervous or psychiatric problems, rheumatoid arthritis, osteoarthritis, or other rheumatism, chronic kidney disease, and asthma. Physical activity was assessed in all waves, except for wave 3, and was based on two questions about the level of daily life physical activity: “How often do you engage in vigorous physical activity, such as sports, heavy housework, or a job that involves physical labour?” and “How often do you engage in activities that require a low or moderate level of energy, such as gardening, cleaning the car, or walking?”. Answers were based on a 4-point scale (1, more than once a week; 2, once a week; 3, one to three times a month; 4, hardly ever, or never). In this study, we used the baseline level of physical activity to classify participants as low (i.e. not doing any activity more than once a week) or high.

### Statistical analyses

The statistical analysis was carried out in two stages, in accordance with the two objectives (Fig. 1). The first objective was to describe and identify clusters in the employment trajectories of men and women. To do this, we used sequence analysis. Sequence analysis is a statistical method for describing the patterns and dynamics of trajectories coded as sequences, i.e. an ordered set of states (status). This approach is commonly used in the social sciences to analyse intensive longitudinal data when the temporal ordering of states needs to be understood. Technically, it is based on methods developed to study DNA, RNA or protein sequences in biology. Sequence analysis in the social sciences focuses on sequences of individual-level trajectories. Each sequence consists of a series of discrete states of the individuals occurring at different points in time. For example, sequences might represent the educational trajectory of young adults, or the occupational trajectory of middle-aged and older workers, or the trajectory of health behaviours over a life course period. It involves collecting information in each year of the trajectory of interest.

Sequence analysis typically involves several steps. First, the sequences are encoded in a standardised format, such as a matrix or string of symbols, where each element represents a particular state. The next step is to align the sequences. In our case, we aligned sequences from age 16 to age 65, so that each participant was assigned a sequence with a length of 49 states. Sequence analysis then involves comparing sequences to identify similarities and differences. We relied on the Hamming distance algorithm^115^ in TraMineR^116^. This distance is known to be sensitive to *timing* and therefore well suited to the analysis of employment trajectories, as employment status is expected to have different effects depending on when it occurs in the life course: for example, being unemployed at age 25 does not have the same implications as being unemployed at age 55. In determining the number of clusters^117^, priority was given to the “Average Silhouette Width” (ASW) measure^118^. Finally, similar sequences were grouped using cluster analysis based on the partitioning around medoids (PAM) algorithm. A cluster represented an employment trajectory with a typical, representative pattern.

For the second objective, we assessed the associations of employment trajectory clusters with the risk of cancer over the life course. To this end, we used logistic regression. Dates of diagnosis covered the whole life course (Figures S1 and S2 in Supplementary materials). All models were adjusted for the following covariates: age, birth cohort, body mass index at baseline, smoking at baseline, number of chronic conditions at baseline, and physical activity at baseline. In this study, baseline was defined as the first wave in which an individual participated in SHARE and completed the prospective questionnaire. Odds ratios (OR) and 95% confidence intervals were estimated. Models were also adjusted with a categorical variable representing participant attrition in the cohort (no dropout, dropout, death). Analyses were run for self-reported overall cancer, and for breast cancer in women. We did not run analyses for other cancer sites due to low prevalence rates (e.g., cervix, colorectal, lung, skin, ovarian, stomach). Statistical analyses were performed with R version 4.3.0^119^.

## Figures and Tables

**Table 1:** Women and men characteristics at baseline, Survey of Health, Ageing, and Retirement in Europe (SHARE), 2004–2020.

	Women			Men		
	All 6,809 women	No cancer (n = 6,028)	Diagnosed with cancer (n = 781)	All 5,716 men	No cancer (n = 4,889)	Diagnosed with cancer (n = 827)
Age at baseline, years (SD)	70.6 (7.0)	70.7 (7.0)	70.1 (6.7)	70.0 (6.5)	70.0 (6.6)	69.9 (6.3)
Birth cohort						
No crisis/war	1387 (20.4)	1,236 (20.5)	151 (19.3)	999 (17.5)	857 (17.5)	142 (17.2)
War period	2367 (34.8)	2,086 (34.6)	281 (36)	2030 (35.5)	1,743 (35.7)	287 (34.7)
Great depression	3055 (44.9)	2,706 (44.9)	349 (44.7)	2687 (47.0)	2,289 (46.8)	398 (48.1)
Attrition						
No dropout	3043 (44.7)	2,651 (44)	392 (50.2)	2340 (40.9)	1,951 (39.9)	389 (47)
Dropout	2098 (30.8)	1,929 (32)	169 (21.6)	1713 (30.0)	1,524 (31.2)	189 (22.9)
Deceased	1668 (24.5)	1,448 (24)	220 (28.2)	1663 (29.1)	1,414 (28.9)	249 (30.1)
BMI, kg/m^2^ (SD)	26.5 (4.5)	26.5 (4.5)	26.6 (4.7)	26.8 (3.6)	26.8 (3.6)	26.8 (3.6)
Smoking status at baseline						
Non-smoker	6150 (90.3)	5,459 (90.6)	691 (88.5)	4740 (82.9)	4,047 (82.8)	693 (83.8)
Smoker	659 (9.7%)	569 (9.4)	90 (11.5)	976 (17.1)	842 (17.2)	134 (16.2)
No. of chronic conditions						
< 2	3543 (52.0)	3,182 (52.8)	361 (46.2)	3524 (61.7)	3,049 (62.4)	475 (57.4)
≥ 2	3266 (48.0)	2,846 (47.2)	420 (53.8)	2192 (38.3)	1,840 (37.6)	352 (42.6)
Physical activity						
Engage in any activity more than once a week	4644 (68.2)	4,096 (67.9)	548 (70.2)	4298 (75.2)	3,672 (75.1)	626 (75.7)
Did not engage in any activity more than once a week	2165 (31.8)	1,932 (32.1)	233 (29.8)	1418 (24.8)	1,217 (24.9)	201 (24.3)
Women’s employment trajectory						
Mainly full-time	1950 (27.7)	1,649 (27.4)	266 (34.1)	-	-	-
Mainly home/family	1662 (23.6)	1,507 (25)	143 (18.3)	-	-	-
Mainly full-time to home/family	1285 (18.3)	1,149 (19.1)	152 (19.5)	-	-	-
Mainly self-employment	583 (8.3)	520 (8.6)	37 (4.7)	-	-	-
Full-time or home/family to part time	624 (8.9)	435 (7.2)	89 (11.4)	-	-	-
Home/family to full-time	433 (6.2)	334 (5.5)	40 (5.1)	-	-	-
Mainly unemployment	95 (1.4)	90 (1.5)	9 (1.2)	-	-	-
Other	398 (5.7)	344 (5.7)	45 (5.8)	-	-	-
Men’s employment trajectory						
Mainly full-time	-	-	-	4691 (82.1)	3,984 (81.5)	707 (85.5)
Mainly self-employment	-	-	-	1025 (17.9)	905 (18.5)	120 (14.5)
Self-reported site of cancer						
Lung	-	-	27 (3.5)	-	-	66 (8)
Colon	-	-	105 (13.4)	-	-	126 (15.2)
Skin	-	-	88 (11.3)	-	-	97 (11.7)
Breast	-	-	353 (45.2)	-	-	10 (1.2)
Cervix	-	-	56 (7.2)	-	-	-

Notes: Data are *n (%)* unless indicated; *BMI* body mass index, *SD* standard deviation

**Table 2:** Associations of women’s employment trajectories from age 16 to 65 with cancer (all sites, n = 781) over the life course. Survey of Health, Ageing, and Retirement in Europe (SHARE), 2004–2020

Types of employment trajectories	OR (95%CI)
Mainly home/family	Ref.
Mainly full-time	1.73 (1.40–2.16)
Mainly full-time to home/family	1.51 (1.19–1.93)
Mainly self-employment	0.76 (0.51–1.09)
Full-time or home/family to part time	2.28 (1.70–3.05)
Home/family to full-time	1.23 (0.84–1.77)
Mainly unemployment	1.05 (0.48–2.03)
Other	1.47 (1.02–2.09)

Notes: Odds ratio (OR) and 95% confidence intervals (CIs); Ref.= reference category. All models are adjusted with covariates (age, birth cohort, BMI at baseline, smoking at baseline, number of chronic conditions, and physical activity) and attrition (reason for dropout).

## Data Availability

This SHARE dataset is available at http://www.share-project.org/data-access.html.
